# In-Line Measurement of Water Content in Ethanol Using a PVA-Coated Quartz Crystal Microbalance

**DOI:** 10.3390/s140101564

**Published:** 2014-01-16

**Authors:** Byoung Chul Kim, Takuji Yamamoto, Young Han Kim

**Affiliations:** 1 Department of Chemical Engineering, Dong-A University, Pusan 604-714, Korea; E-Mail: kbc1010@hanmail.net; 2 Department of Chemical Engineering, University of Hyogo, Himeji 671-2201, Japan; E-Mail: tyamamot@eng.u-hyogo.ac.jp

**Keywords:** quartz crystal microbalance, water content in ethanol, PVA-coated sensor

## Abstract

An in-line device for measuring the water content in ethanol was developed using a polyvinyl alcohol (PVA)-coated quartz crystal microbalance. Bio-ethanol is widely used as the replacement of gasoline, and its water content is a key component of its specifications. When the PVA-coated quartz crystal microbalance is contacted with ethanol containing a small amount of water, the water is absorbed into the PVA increasing the load on the microbalance surface to cause a frequency drop. The determination performance of the PVA-coated microbalance is examined by measuring the frequency decreases in ethanol containing 2% to 10% water while the ethanol flows through the measurement device. The measurements indicates that the higher water content is the more the frequency reduction is, though some deviation in the measurements is observed. This indicates that the frequency measurement of an unknown concentration of water in ethanol can be used to determine the water content in ethanol. The PVA coating is examined by microscopy and FTIR (Fourier transform infrared) spectroscopy.

## 1. Introduction

The prospective use of ethanol for automobile applications is expected to increase steadily, because as a fuel it is relatively clean and sustainable. Though chemical synthesis has been a major process for its production, the production from grain fermentation is desirable due to its sustainability. The beer produced from the fermentation has low ethanol contents, and it is concentrated to a fuel quality ethanol using conventional separation processes. In the concentration process the in-line measurement of the water content in ethanol is necessary for the process control, and a simple in-line measurement technique is preferred to help the practical operation of the concentration process.

Various sensor technologies have been applied to determine water content in ethanol for in-line applications. Though many instruments have been developed to measure the water content, the in-line measurement is more convenient to apply to the ethanol concentration process. One off-line technique was introduced by Bruno *et al.* [1]. They measured the water content from the distillate volume fraction obtained from a standard distillation apparatus. Because optical methods are easy to use for in-line applications, many such techniques have been proposed and tested. Fujiwara *et al.* [2] used a laser as the light source, and measured the reflection intensity of the applied laser to determine the water content. Another optical technique measured the near IR absorbance to measure the water content [3]. In another study ultrasound was applied to the sample, and its velocity was measured to calculate the water content in commercial beverages [4]. Because of its easy in-line applicability high frequency electric signals were applied to a coaxial stub immersed in the flow line of a liquid sample [5]. The dielectric permittivity of the sample was measured from the variation of amplitude, which can be used to determine the ethanol and water composition. These techniques utilize large instruments and require sophisticated peripheral accessories.

A quartz crystal microbalance (QCM) has a thin quartz crystal plate with two metal electrodes on both sides that establish an alternating electric field across the plate, causing vibration of the plate at its resonant frequency. The frequency is sensitive to mass loading and micro-rheology on the electrodes. The QCM utilizes the characteristic of frequency variation with the changed mass loading and visco-elasticity [6]. The change of mass loading due to crystal formation has been used to detect the moment of crystal nucleation [7] and growth [8–11]. The change was also applied to detect the dew point of organic vapors from the condensation on the electrode surface [12]. From the variation of micro-rheology the kinetic rate of photopolymerization was measured to analyze polymerization kinetics [13,14]. The thermal properties of polymers were also measured from the change of visco-elasticity of the QCM [15,16].

Instead of the direct measurements of surface loading, a modification of the electrode surface of a quartz crystal microbalance has been utilized for the entrapment of target materials inducing a load change. In the determination of organic substances either in gas or liquid phase, organic films have commonly been coated on the surface of one of its electrodes [17–19]. Though many polymer film-coated sensors [20–22] have been applied to various applications with improved stability of the coated material and selectivity of the detected material, no application to the measurement of water content in ethanol has been reported. Recently the QCM has been widely used in bio-applications [23,24]. The effect of water in the measurement of toluene dissolved in water using a polymer coated QCM was examined by Pejcic *et al.* [25,26], and it was found that the water exposure time has a significant effect on sensitivity at high toluene concentration. Pejcic *et al.* [27] also found that the molecular selectivity of a polymer can be improved by the proper plasticizer applied to the polymer- coated QCM for the measurement of aromatic hydrocarbons in water.

In this study, a measurement device for water contents in ethanol is developed by applying a PVA thin film on the electrode surface of the QCM. For the performance evaluation, 2% to 10% water contents are utilized to determine the frequency drop of the polymer film-coated QCM. The measurement is conducted in a flow system of the ethanol samples. In addition, the characteristics of the film are investigated by microscopic observation and FTIR analysis.

## 2. Experimental Section

### 2.1. Materials

Polyvinyl alcohol (Mw. 22,000) was obtained from Junsei Chemical (Tokyo, Japan), and ethyl alcohol (99.99%) was from Burdick & Jackson (Ulsan, Korea). The chemicals were used as received. Quartz crystal microbalances (Sunny Electronics Co., Chungju, Korea) were purchased from a local store in capped state for electronic circuit use. The cap was removed before coating the polymer films.

### 2.2. Analytical Instruments

Spectroscopic analysis was conducted with an FT-IR spectrometer (Model Nicolet 380, Thermo Scientific, Waltham, MA, USA), and microscopic observation was done with a scanning electron microscope (Model JSM 6700F, JEOL Ltd., Tokyo, Japan,) and a microscope (Model iMegascope, Sometech, Seoul, Korea).

### 2.3. Equipment

**Figure 1.**
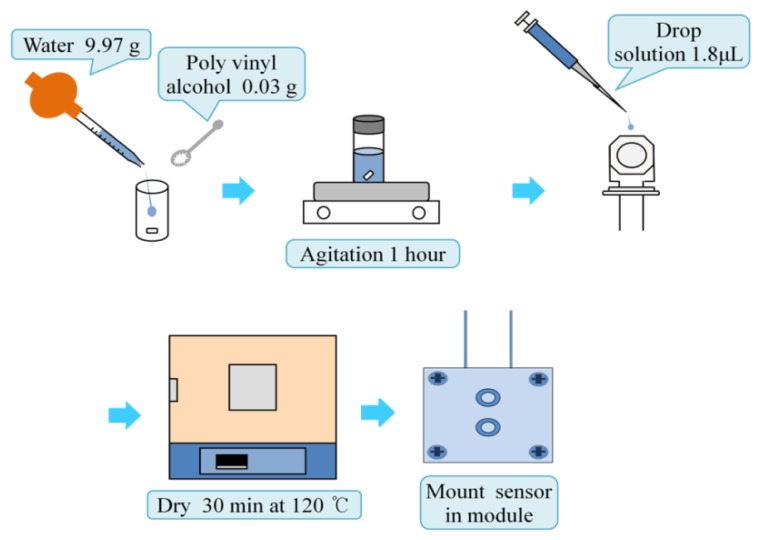
Flowchart of the preparation procedure of PVA coated quartz crystal microbalance.

An AT-cut quartz crystal microbalance having a base frequency of 8 MHz was utilized in this experiment. The electrodes of the microbalance were silver finished. The microbalance was rinsed with ethanol before applying the PVA. The PVA was dissolved in water at a concentration of 0.3%, and the solution was stirred for an hour. A 1.8 μL sample of the completely dissolved PVA solution was spread on the one of microbalance electrode surfaces. After briefly drying in air, the microbalance was baked in an oven at a temperature of 120 °C for half an hour. The microbalance was cooled in a Petri dish containing silica gel for 10 min. The PVA thin film was coated twice following the same procedure. Figure 1 describes the sensor preparing procedure. The cell module holding the microbalance for the batch experiment was built with polypropylene plates. The thicknesses of two plates were 5 mm. The top plate has a hole to make one electrode of the microbalance contact with the ethanol, and the other is sealed from the ethanol. To prevent leakage a thin silicone gasket and two o-rings are placed between the two plates. The size of the two plates was the same with a dimension of 30 mm by 50 mm. Six screws tighten the two polypropylene plates.

**Figure 2.**
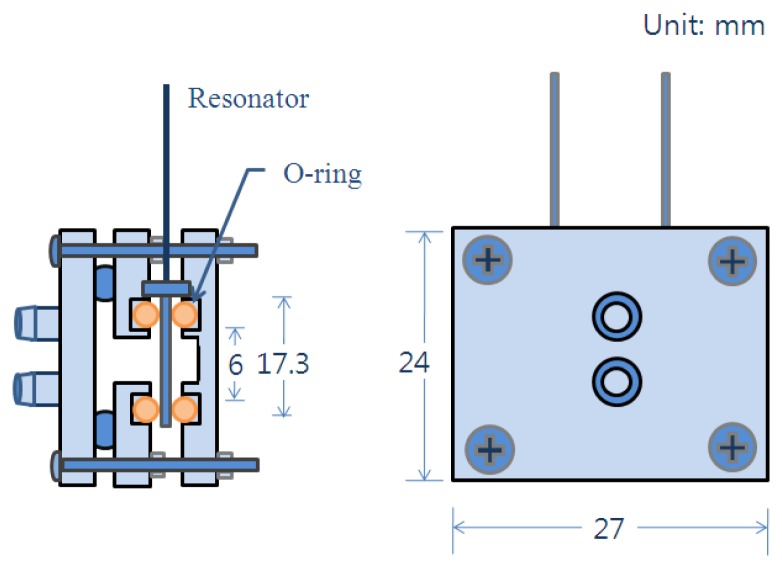
Schematic diagram of the holding cell of quartz crystal microbalance for in-line measurement.

**Figure 3.**
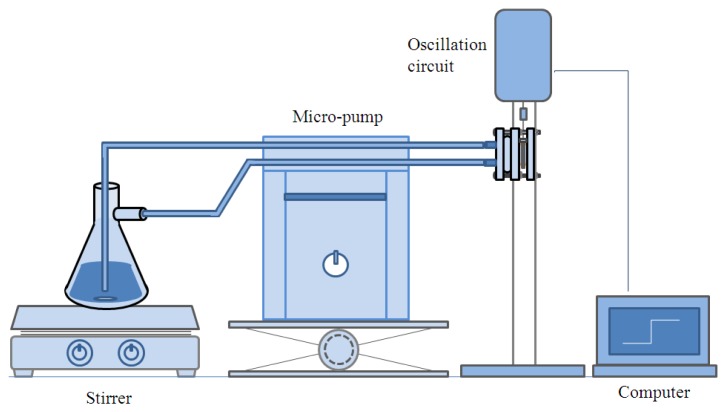
Schematic diagram of experimental setup for in-line measurement.

For the in-line measurement the quartz crystal microbalance was installed in the module as described in Figure 2, and the module was placed in the experimental setup as shown in Figure 3. The ethanol was flow through the two nipples prepared on the left hand side plate of the cell module.

### 2.4. Procedures

The measurements of water content in ethanol were conducted in a beaker for batch measurement and in the flow system for in-line measurement. The procedures are explained separately as below.

#### 2.4.1. Batch Measurement

The PVA-coated microbalance was mounted in the cell module, and placed vertically in a 50 mL beaker containing ethanol. While the ethanol was stirred mildly, the resonant frequency was measured. The oscillation circuit provides a small amount of oscillating current to the microbalance, and the resonant frequency of the microbalance is determined by the load and rheological variation on the surface of the microbalance electrode. The frequency is counted with a home-made counter connected to a PC. The measured frequency was stored in the PC for later data analysis. When the frequency is stable, water is added to ethanol to increase the water content by 2 wt% each time up to 10%.

#### 2.4.2. In-Line Measurement

After the microbalance was placed in the cell module as described in Figure 2, the experimental setup was assembled as illustrated in Figure 3. While the ethanol flowed through the module at a rate of 10 mL/min, the variation of resonant frequency was measured continuously with the PC. The water content was adjusted by adding distilled water in the ethanol for the increase, and ethanol was added for the decrease of water content. The experiment was conducted from 2 wt% of water to 10 wt% with a 2% increase in each step for about 4 min, then the content was decreased by 2 wt% in each step down to 2 wt%.

## 3. Results and Discussion

The experimental results of batch and in-line measurements were analyzed separately, and the PVA coating was characterized.

### 3.1. Batch Measurements

**Figure 4.**
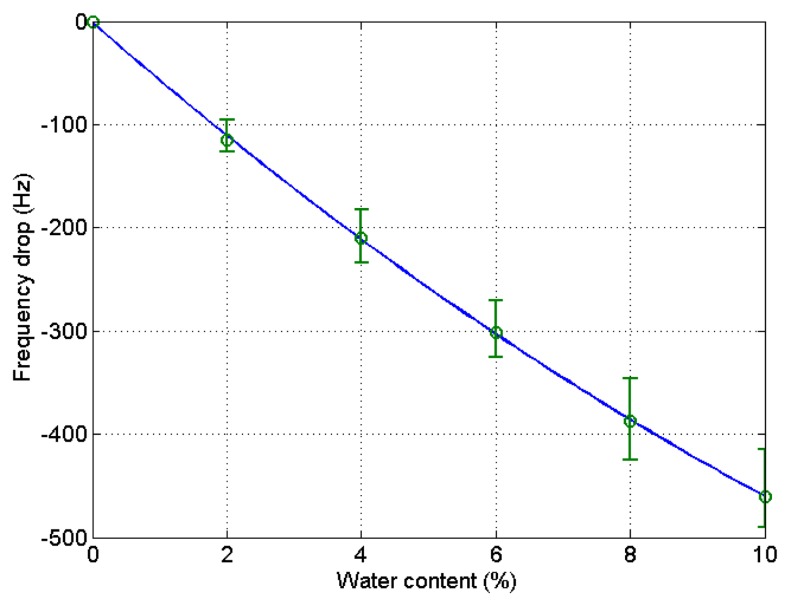
Relationship between water content and resonant frequency shift of bare quartz crystal microbalances in batch measurement.

After the experiment was over, the stored data was retrieved from the PC to analyze the experimental outcome. The drift at the 6% and 8% water content in the blank microbalance measurement was caused by the incomplete mixing of the solution. The change of solution density and viscosity causes the frequency variation. In order to eliminate this blank change from the actual measurement the blank test was conducted. The step-wise decrease of the frequency indicates the effect from the elevation of water content by 2% each. The measurement was continued for about 8 min at each water contents until the frequency stabilized. The test was carried out four times, and the results are demonstrated in Figure 4. A fitted curve was derived from the data to be used for the compensation of the frequency variation of the blank microbalance. Though the fitted curve is slightly bent, the frequency drops almost linearly with the increase of the water content. The correlation coefficient (*R*^2^) was 0.98.

**Figure 5.**
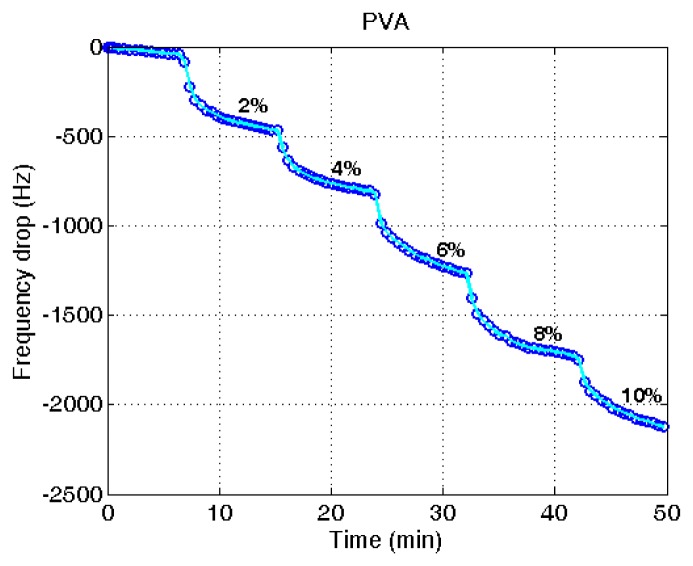
Variation of resonant frequency of a PVA-coated quartz crystal microbalance with increased water content.

**Figure 6.**
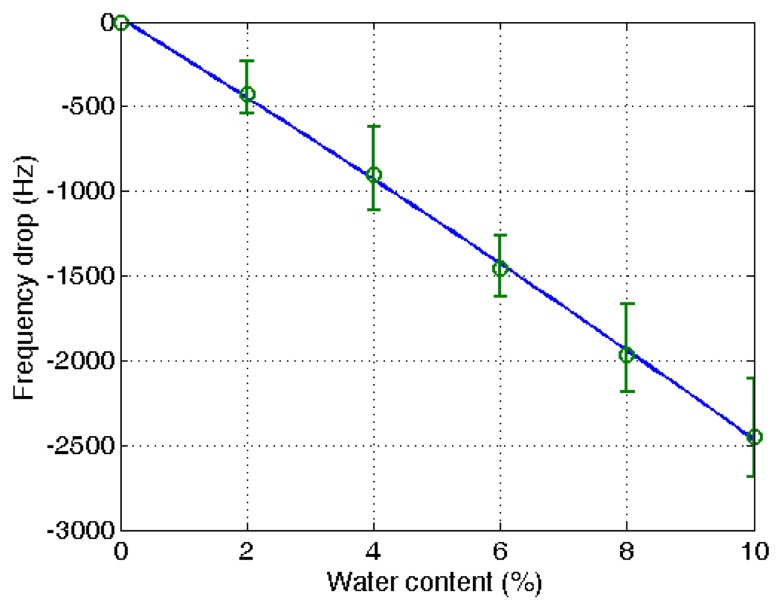
Relationship between water content and resonant frequency shift of PVA-coated quartz crystal microbalances.

Figure 5 shows typical measurements of the frequency variation at different water contents in ethanol. Each measurement was taken after 8 min from the water addition to the ethanol. The measurements demonstrate the steady decrease of the frequency with the raised water content. Though the frequency drop was continued after 8 min of measuring time, the drop was significantly reduced after the measuring time. In the application of the microbalance for the in-line measurement used for process control, the measuring time is limited and therefore the time was set at the 8 min. From the eight repeated measurements of the frequency variation, a combined plot was obtained, as given in Figure 6. The measurements were adjusted with the amount of PVA applied after the blank deduction was counted. The PVA amount was measured with the frequency drop before and after the PVA coating.

A linear decrease of the frequency with the elevated water content was obtained (*R*^2^ = 0.97). It indicates that the unknown water content in ethanol can be determined from the measured reduction of the resonant frequency using the proposed QCM sensor system. Though there is some deviation with different microbalances, the decrease of the frequency with the increased water content is demonstrated. The measurement of water content using a quartz crystal microbalance is relatively simple compared with other measurement techniques utilizing analytical instruments.

### 3.2. In-Line Measurements

**Figure 7.**
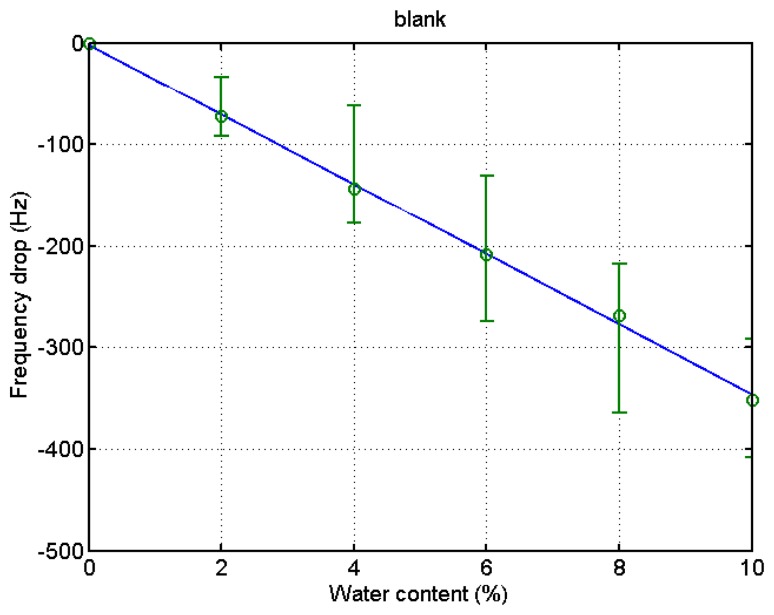
Relationship between water content and resonant frequency shift of a bare quartz crystal microbalance with increased water content in an in-line measurement.

For the in-line measurement of water content in ethanol, the experiment was conducted in the flow system as shown in Figure 3. When the water content increases, the frequency is reduced. Then, the frequency recovers as the water content decreases. It indicates that the measurement with the PVA-coated microbalance needs to compensate for the blank effect. For the compensation a linear relation of the blank effect is derived from four blank microbalance tests as shown in Figure 7 (*R*^2^ = 0.85).

**Figure 8.**
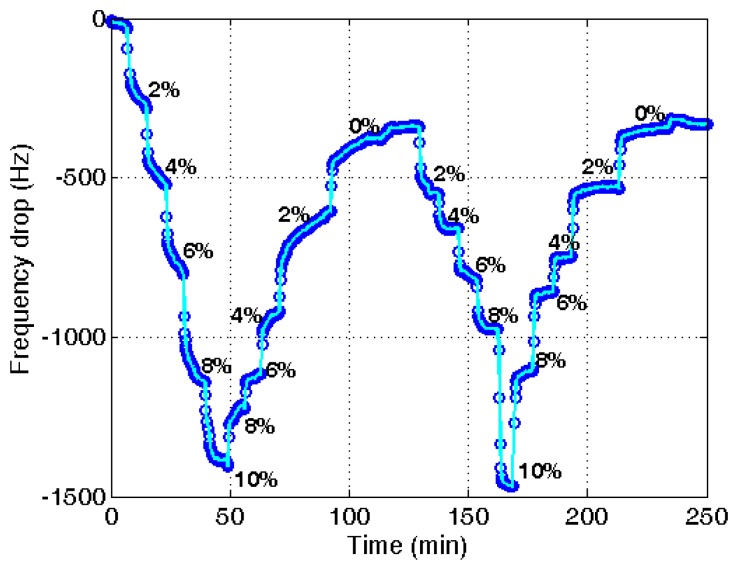
Variation of resonant frequency of a PVA-coated quartz crystal microbalance with increased water content in an in-line measurement.

**Figure 9.**
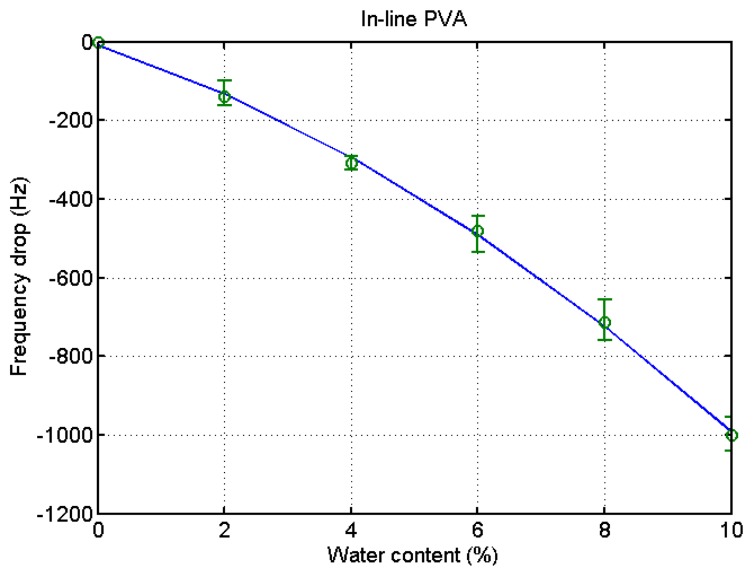
Relationship between water content and resonant frequency shift of a PVA-coated quartz crystal microbalance with increased water content in an in-line measurement.

The same procedure was applied to the ethanol of different water contents using a PVA-coated microbalance. Figure 8 shows the frequency variation with water content increase and decrease. The variation is similar to that of the blank microbalance. Note that the blank effect is deduced from the measurement. From the three measurements of water content using the PVA-coated microbalance a curve is derived as shown in Figure 9 (*R*^2^ = 0.99). There is some deviation, but the variation is large enough to find the water content from the measurement of unknown samples in an in-line application. While the resonant frequency drop with a bare quartz crystal microbalance is 460 at 10% water content as shown in Figure 4, that of the PVA coated microbalance is 2450 as illustrated in Figure 6. The sensitivity of the PVA-coated microbalance is improved 5.3 times compared with the bare microbalance. In case of in-line measurements the sensitivity was improved 2.9 times. In this experiment the availability of the quartz crystal microbalance for the water content determination was proved for the batch and in-line measurements. The variation of the resonant frequency with different water contents in the in-line measurement is shown in Figure 8. The frequency was not fully recovered with the reduced water content, but the recoveries in the first and second cycles are similar. It indicates that when the sensor is weathered its recovery does not affect the measurement of water content. The low recovery is due to the binding between water and the PVA coating. Buslo *et al.* [28] investigated the binding between water molecules and PVA via IR spectroscopy, and found that the water molecules form hydrogen bonds with the hydroxyl groups of the PVA.

### 3.3. PVA Characterization

**Figure 10.**
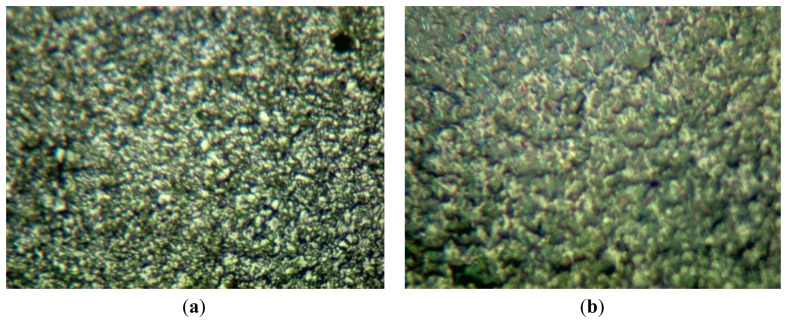
Photographs of optical microscope of microbalance surface magnified by 1200 times: (**a**) blank; and (**b**) PVA coated.

**Figure 11.**
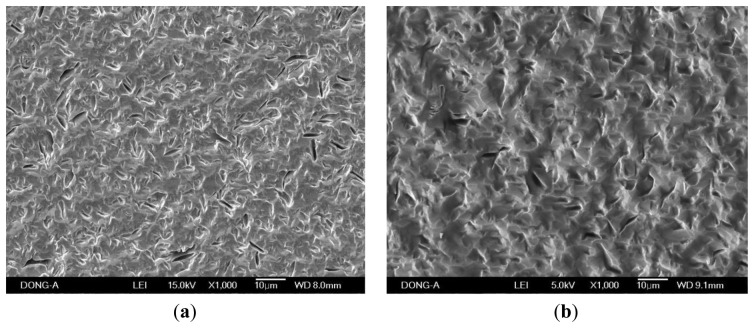
Photographs of scanning electron microscope of microbalance surface magnified by 1000 times: (**a**) blank; and (**b**) PVA coated.

**Figure 12.**
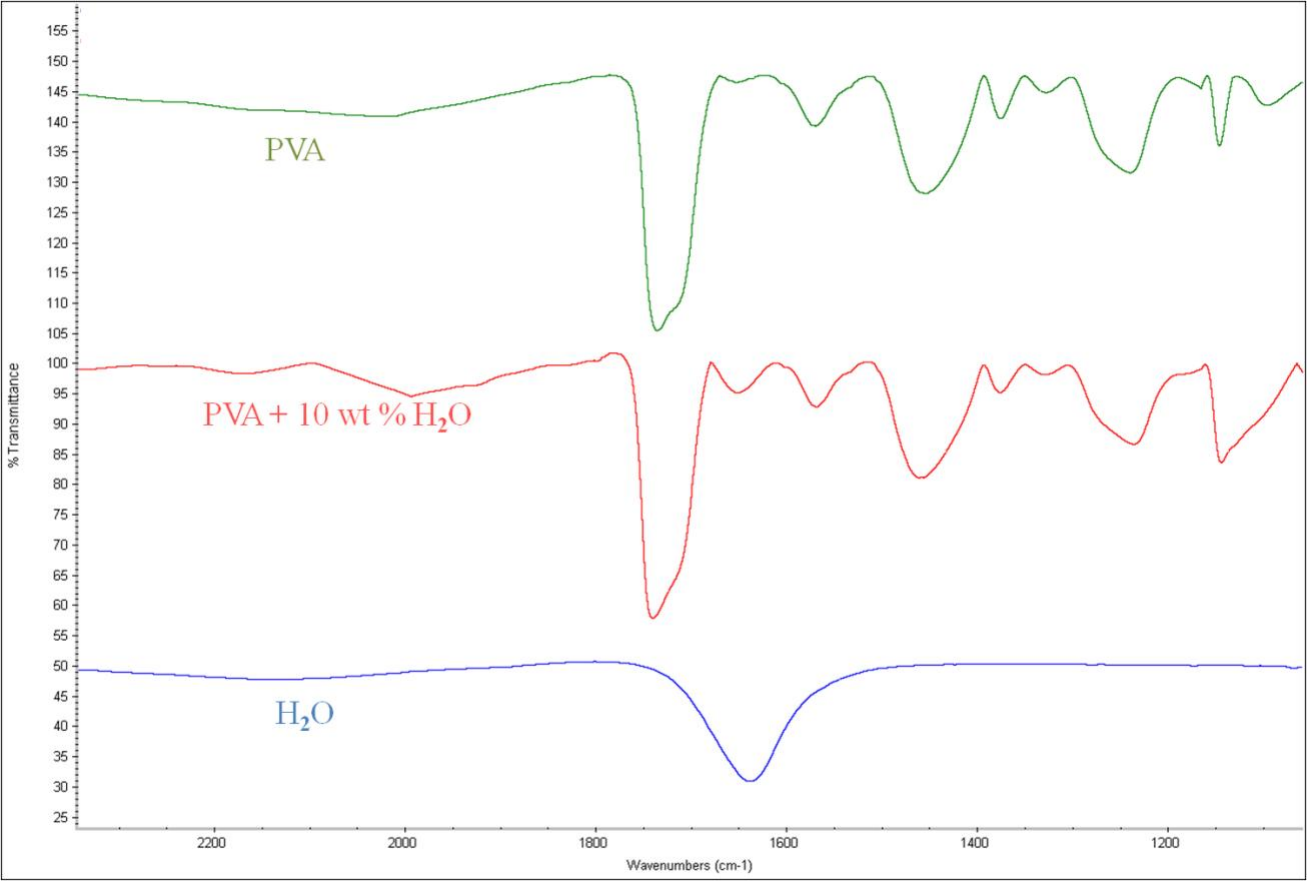
FTIR spectra for the comparison of PVA with and without water.

When the PVA was applied to the microbalance surface, it was coated evenly on the surface. The amount of PVA coating affects the frequency variation, and therefore the measurements were calibrated for the amount of the coating. The coating amount was determined from the difference between the resonant frequencies before and after the coating. Figure 10 illustrates the microscopic observation of the surface. The left figure shows the surface of a bare microbalance, and the right one shows the PVA-coated microbalance. The PVA coating spread on the surface is seen from the disappearance of small holes shown in the bare microbalance surface. A similar observation was found from the scanning electron microscope photograph given in Figure 11. Again the left corresponds to the bare microbalance magnified by 1000 times, and the right picture is the photograph of the PVA-coated microbalance. The PVA coating is demonstrated in the right picture. The binding of water and PVA was measured with an FTIR spectrometer, and the resulting spectra are shown in Figure 12. When the spectra of PVA only and PVA and water are compared at the wavelength of water peak in the bottom spectrum, there is a higher peak in the spectrum of PVA and water at the water wavelength. The comparison indicates the binding of PVA and water, which is used in the measurement of water content of this study.

## 4. Conclusions/Outlook

A simple device using a quartz crystal microbalance to determine water content in ethanol was proposed here. The quartz crystal microbalance was coated with PVA for the improved sensitivity with water, and its performance was examined with samples of known water content in batch and flow systems. The results calibrated with blank deduction indicate that there is strong relation between the resonant frequency drop and water content. This outcome can be utilized in the determination of unknown water content in ethanol by measuring the frequency of the PVA-coated QCM for the unknown sample. The measurement results in the flow system prove that the proposed microbalance can be used for in-line applications. The condition of the PVA coating was investigated by optical and scanning electron microscopy and FTIR spectroscopy.
